# Direct Fabrication of Functional Shapes on 3D Surfaces Using Electrospinning

**DOI:** 10.3390/polym15030533

**Published:** 2023-01-20

**Authors:** Ioana Caloian, Jocelyn Trapp, Melissa W. Williams, Ryan A. Kim, Mahmoud E. Moustafa, Eva Hawa Stwodah, Christina Tang

**Affiliations:** 1 Department of Chemical and Life Science Engineering, Virginia Commonwealth University, Richmond, VA 23284-3028, USA; 2 Department of Fashion Design and Merchandising, Virginia Commonwealth University, Richmond, VA 23284-2519, USA

**Keywords:** electrospinning, nanofibers, patterned fibers, template assisted, self-assembly, fabric handle

## Abstract

In this work, we demonstrate the ability to simultaneously pattern fibers and fabricate functional 2D and 3D shapes (e.g., letters, mask-like structures with nose bridges and ear loops, aprons, hoods) using a single step electrospinning process. Using 2D and 3D mesh templates, electrospun fibers were preferentially attracted to the metal protrusions relative to the voids so that the pattern of the electrospun mat mimicked the woven mesh macroscopically. On a microscopic scale, the electrostatic lensing effect decreased fiber diameter and narrowed the fiber size distribution, e.g., the coefficient of variation of the fiber diameter for sample collected on a 0.6 mm mesh was 14% compared to 55% for the sample collected on foil). Functionally, the mesh did not affect the wettability of the fiber mats. Notably, the fiber patterning increased the rigidity of the fiber mat. There was a 2-fold increase in flexural rigidity using the 0.6 mm mesh compared to the sample collected on foil. Overall, we anticipate this approach will be a versatile tool for design and fabrication of 2D and 3D patterns with potential applications in personalized wound care and surgical meshes.

## 1. Introduction

Electrospinning is a simple and versatile method for producing nanofibers and microfibers from a variety of functional materials with potential applications in filtration, tissue engineering, drug delivery, electronics, etc. [1,2,3]. To electrospin fibers, a voltage is applied to a capillary containing a polymer solution or melt. The potential difference between the capillary and the grounded collector leads to electrostatic stress that overcomes surface tension ultimately resulting in a continuous jet that travels from the tip of the capillary to the collector. As the jet travels to the collector, it is whipped and stretched and the solvent rapidly evaporates. Ultimately, the fibers are deposited randomly and collected as a nonwoven mat [4,5,6]. The ability to pattern fibers by controlling the deposition of fibers during electrospinning would have important implications for applications such as filtration, electronics, and tissue engineering [3,7,8]. For example, for filtration applications, oriented fibers are desirable for reducing pressure drop and enhancing filtration efficiencies [9]. 

While single nozzle electrospinning is a well-established technique, there are ongoing efforts to scale up electrospinning processes with respect to mass throughput. Techniques such as needle-free electrospinning, bubble electrospinning, and wire spinnerets have been used to accelerate fiber production via electrospinning [10,11,12]. These techniques have involved adapting the configuration of the spinneret. Industrial electrospinning equipment based on multiple jet or various spinneret geometries (cylinder, disk, wire) with throughput of 300–600 g/h are available [11]. Commercially, this is a well-established process for air filtration products [11] and battery separators [1]. Emerging applications that are transitioning from lab to commercial scale are tissue scaffolds for tendon repair, sound absorption materials, face masks, and multifunctional clothing [10].

Complementary to approaches to adapt the spinneret to increase throughput, techniques to adapt the collector have facilitated manipulation of fiber deposition. For example, aligned fibers can be achieved by using gap electrospinning across two parallel electrodes (conductive) or by using a rotating collector such as a copper wire collector [2,9]. Near-field electrospinning has also been used to control fiber deposition and enable direct writing of highly aligned and complex microarchitectures [2,8]. Magnetic field-assisted electrospinning (permanent magnets introduced into the electrospinning set-up) has also been used to manipulate the deposition of fibers and achieve aligned fibers [2]. Use of dielectric materials for gap collectors has also facilitated control of the electric field profile. Yan et al. demonstrated that the relative permittivity of the collector was a key factor in controlling fiber deposition [13] and aligned fibers were achieved [14]. Liquid collectors have also been used to align fibers [14].

Building on these techniques to manipulate fiber deposition, patterning of nanofibers has been achieved using various classes of collectors (conductive, liquid, etc.) to template fiber deposition. Patterned conductive electrodes result in a locally concentrated electric field that attract electrospun fibers; protrusions in the collector template facilitate patterning of the deposited fibers [15]. By placing patterned, non-conductive templates on foil, emblems, and various patterned architectures were demonstrated [16]. Complex templates based on printed circuit chips have also used been as templates for electrospun nanofiber assembly and to produce fiber mats with complex shapes, e.g., alphanumeric characters (1mm × 1mm) [17]. 

Electrolyte solutions (e.g., 1 M KCl, selectively patterned on dielectric polymer substrates) have also been used to template patterned electrospun fibers. Nanofiber mats with complex shapes such as stars, hexagonal arrays, and emblems were made using this approach and used to align cells in vitro [18]. Periodic fiber arrays (features 50–400 microns) were fabricated using a patterned pyroelectric lithium niobate crystal as a collector and applying heat to create a bipolar electric field (with negative polarity on the walls of the collector and positive polarity in the gaps of the collector) [7]. Although these methods produce exemplary results, they require complicated fabrication of prepatterned collectors, masks, and molds [7]. Further, while free-standing electrospun mats have been produced, shapes have been limited to millimeter to centimeters. Limitations in area when fabricating aligned and patterned fibers remains a challenge [2]. 

Alternatively, the use of various conductive mesh collectors has enabled facile fabrication of highly ordered fibrous mats [19]. The effect of collector geometry on functional fibrous mat properties is an emerging area. For example, the effect of collector geometry on pore size [20], filtration efficiency [21,22], optical transparency [22], wettability [22], and tensile strength [20,21,22] have been examined for tissue scaffold [20] and filtration applications [21,22]. For broader applications, e.g., wearables and wound healing, the ability to combine patterning and the ability to make customizable shapes have yet to be demonstrated. Further, the effect of patterning on functional properties such as rigidity of the fiber mat affecting “handle” of the material has not been fully established.

In this work, we combine fiber patterning and fabrication of complex 2D and 3D shapes into a single step electrospinning processing. Conductive wire meshes were formed into templates of desired functional shapes and used directly as collectors for electrospinning. The assembly of the fibers on the length scale of the mesh and the template were investigated. The effect of mesh size on functional properties, i.e., wettability and rigidity (quantified by flexural rigidity), was examined. The ability to achieve functional 2D and 3D shapes is demonstrated.

## 2. Materials and Methods

Nylon-6 pellets (ULTRAMID B40 01) were obtained from BASF Corporation (Wyandotte, MI, USA) and used as received. Formic acid (reagent grade) was received from Sigma-Aldrich (Milwaukee, WI, USA) and used as received. 

For electrospinning, 20 wt.% nylon was dissolved in formic acid by stirring at room temperature until macroscopically homogenous. The solution was then electrospun using a point-plate configuration composed of a precision syringe pump (New Era NE-300: Farmingdale, NY, USA) and a high-voltage power supply (Matsusada Precision Inc., model AU-40R0.75 with positive polarity, Kusatsu, Shiga, Japan) with positive polarity as previously described [23]. Typical electrospinning parameters were a flow rate of 0.15 mL/h, tip-to-collector distance of 12 cm, 22-gauge blunt tip (0.508 mm i.d.), and an operating voltage of ∼20 kV. Fibers were collected on a 28 cm plate covered with aluminum foil with a non-stick coating (Reynolds Consumer Products, Lake Forest, IL, USA). Alternatively, fibers were collected on metal wire meshes. Three different size woven wire meshes were used: 30 mesh with a 0.6 mm mesh size and 250 micron wire diameter, 18 mesh with a 0.9 mm mesh size and 400 micron wire diameter, and a ¼’ mesh with a 6.4 mm mesh size and a 650 micron wire diameter. We refer to these substrates by the gap size: 0.6 mm, 0.9 mm, and 6 mm mesh, respectively. Collectors of various sizes and shapes were used. 

The samples were characterized with optical light microscopy (Nikon Eclipse 150N Instruments Inc., Melville, NY, USA). For higher resolution imaging, the samples were sputter-coated with platinum, and the fiber morphology was examined with SEM using Hitachi SU-70 FE-SEM (Tokyo, Japan) (accelerating voltage of 5 kV). The average fiber size and standard deviation were determined by measuring the diameter of 100 fibers using ImageJ software version 1.53 developed by US NIH. The surface hydrophobicity properties of the fiber mats were evaluated by static contact angle measurement using a goniometer (OCA 15, DataPhysics Instruments; Charlotte, NC, USA) at room temperature. The electrospun mats were cut into 25 mm × 25 mm samples and removed from the foil/mesh for testing without any backing layer (e.g., wax paper). A sessile droplet of 5 µL deionized water was used. The flexural rigidity was measured using the heart loop method ASTM 1388 D [24]. Samples approximately 1” × 9” were prepared using the various sized meshes cut to size the sample area (cm^2^) was determined. The mass of each sample was recorded in mg. The ends of the samples were fastened to 2.5 cm wide bars similar to previous reports [25]. A heart-shaped loop was formed and hung vertically under its own weight. Side view images of the heart loop were taken with a ruler in the frame (Supporting Information—Figure S1). Experimentally, the loop length and the loop height were measured (both in cm). The loop length was determined by tracing the length of the loop from one end to the other, and the loop height (i.e., the vertical height from the clamp to the bottom of the loop) was measured using ImageJ software. The measured lengths were used to calculate bending length, *c* in cm, according to:

(1) $$h_{0}=0.1337L$$

(2) $$d=h-h_{0}$$

(3) $$\theta =32.85{}^{\circ}\frac{d}{h_{o}}$$

(4) $$f\left(\theta \right)={\left(\frac{cos\;\theta \;}{tan\;\theta \;}\right)}^{\frac{1}{3}}$$

(5) $$c=h_{0}f\left(\theta \right)$$

Using the bending length (*c* in cm) and the sample weight per unit area (*w* in mg/cm^2^), the flexural rigidity, *G*, was calculated in mg∙cm [24,26]:

(6) $$G=w\times c^{3}$$

Each side of the sample was measured [24]. For each sample, the measurement was performed five times. The average and standard deviation of the trials are reported. 

## 3. Results and Discussion

**Figure 1:**
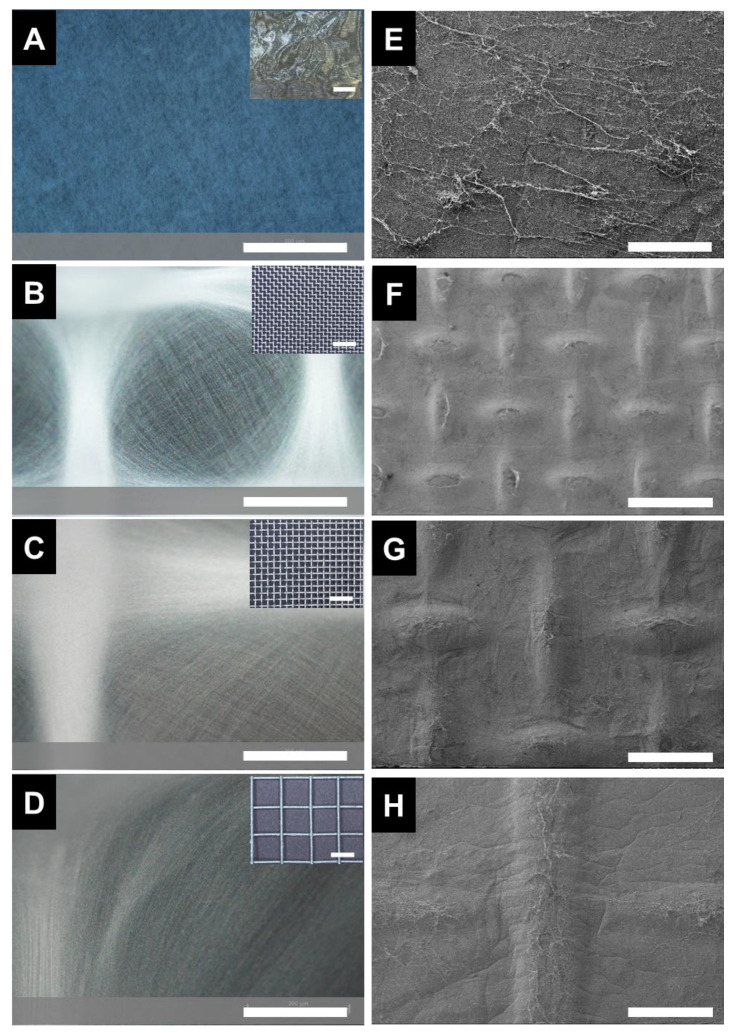
Microscopy of the electrospun samples. Optical micrographs at 10× magnification of samples spun on collected on (**A**) foil, (**B**) 0.6 mesh, (**C**) 0.9 mesh, and (**D**) 6 mm mesh with 200 micron scale bars. The collectors are shown as insets. The scale bars of the insets are 5 mm. SEM micrographs at 30× magnification of samples spun on collected on (**E**) foil, (**F**) 0.6 mesh, (**G**) 0.9 mesh (**H**) 6 mm mesh with 1 mm scale bars.

During the electrospinning process, the fibers generated are typically deposited randomly. Using patterned collectors has enabled control of the organization of fibers [15,20]. To pattern the fibers via self-assembly during fiber processing, we electrospun nylon onto electroconductive collectors with woven structures (comprised of metal protrusions and void spaces). During electrospinning, the fibers preferentially deposited on the metal protrusions relative to the voids. Thus, macroscopically, the pattern of the electrospun mat mimicked the woven mesh (Figure S2). The pattern was consistent over the entire area of the electrospun sample. These results are comparable to previous reports [19]. The preferential assembly of the fibers along protrusions of the mesh relative to the voids were also evident from the optical microscopy images (Figure 1A–D). This result is consistent with previous reports [20] and has been attributed to the electrostatic forces between the fiber and the mesh [15]. Examining the fiber pattern with SEM, the fiber patterning was dictated by the collector used (Figure 1E–H). In contrast, no fiber patterning was observed in the sample collected on foil. Thus, the fiber patterning can be attributed to the effect to Coulombic interactions [10]; specifically, the patterned metal mesh acts as an “electrostatic lens”, focusing deposition onto the conductive walls of the mesh [19].

**Figure 2:**
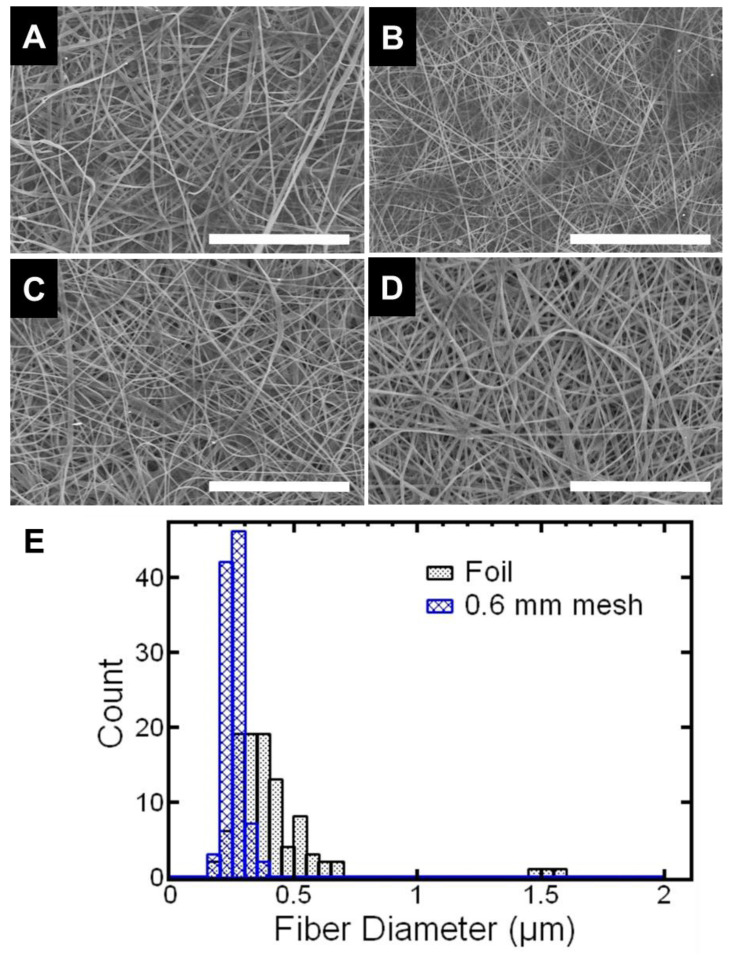
SEM micrographs at 5000× magnification to capture fiber diameter of the electrospun samples collected on (**A**) foil, (**B**) 0.6 mm mesh, (**C**) 0.9 mm mesh, and (**D**) 6 mm mesh. The scale bar represents 20 microns. Patterning generally decreases fiber size and narrows the fiber size distribution. The effect is most apparent when comparing the sample collected on foil and the sample collected on the 0.6 mm mesh. Fiber size distributions of both samples are shown in (**E**).

Next, we investigated the effect of the mesh on the resulting fiber diameter. Fiber diameter was measured from the SEM images (5000× magnification, Figure 2A–D) of each of the samples. We observed that using the woven mesh collector generally decreased fiber diameter and narrowed the fiber size distribution compared to the foil collector. For example, fibers collected on the 0.6 mm mesh had an average fiber diameter of 256 nm ± 36 nm compared to fibers collected on foil with an average fiber diameter of 409 nm ± 223 nm. Thus, the fiber diameters using the mesh were more uniform as quantified by the coefficient of variation. The coefficient of variation of the fiber diameter the 0.6 mm mesh was 14% compared to 55% for the sample collected on foil. The trend in fiber diameter and uniformity were similar but less pronounced with the other meshes (increasing mesh size). For example, the sample on the 0.9 mm mesh had a fiber diameter of 320 nm ± 113 nm with a coefficient of variation of 35% (i.e., larger than the 0.6 mm mesh and smaller than foil) (Table S1—Supporting Information). Similar results have been reported previously [20] and may be attributed to the electrostatic lensing effect. Further, we note that at the size scale of an individual fiber (~micron) the fibers are randomly oriented and at the scale of the mesh (~mm) the fibers self-assemble due to the pattern of the mesh. Overall, fiber patterning generally decreases fiber size and narrows the fiber size distribution. The effect is most apparent when comparing the sample collected on foil and the sample collected on the 0.6 mm mesh.

Next, we investigated the effect of the introduced patterning on the functional properties of the electrospun material, which may be particularly useful for wearable applications and wound healing applications [27,28,29,30]. For such applications, mechanical properties and hydrophobicity are important considerations [31]. The feel or “handle” (or drape) of a fabric is a major consideration [31]. An important mechanical property used to assess the handle of a material is the fabric stiffness quantified by the flexural rigidity. The flexural rigidity physically represents the resistance to bending that would be appreciated by the fingers and can be measured non-destructively [32,33]. Another important functional property would be hydrophobicity For example, materials with water-repellent properties may have applications in protective wear [34]. Water-repellent properties can be determined from the contact angle of the surface with the drop of water [21]. When the contact angle is over 90 degrees, the surface can be considered repellent [35]. Thus, we investigated the rigidity and the hydrophobicity of the patterned fiber samples. 

The effect of patterning on mechanical properties has been previously studied using tensile testing. Notably, the mechanical properties of samples electrospun onto meshes have been compared to samples electrospun onto foil (randomly oriented). When using a comparable mesh (0.8 micron spacing) to the meshes used here, patterned nylon-6,6 showed an increased yield stress compared to randomly oriented fibers (approximately 5-fold higher) at comparable strains [22]. Similar increases in yield stress were observed with nylon-6,6 fibers produced on meshes with other shapes (e.g., herringbone pattern) [21]. Building on this work, we focused on characterizing the mechanical properties of the patterned fiber mat related to the functional feel or “handle” (or drape) of the nonwoven fabric material. The fabric stiffness quantitatively assessed by the flexural rigidity to assess the handle of a material as it physically represents the resistance to bending that would be appreciated by the finger [32,33]. Thus, the effect of the introduced patterning on the flexural rigidity of the samples was characterized. The effect of the introduced patterning on the rigidity of the samples was characterized. The rigidity of the samples was measured using the heart loop method (preferred for soft samples with bending lengths less than 2 cm or samples that tend to curl) [33] to measure the bending the sample under its own weight. From the measurements, the bending lengths and flexural rigidities were calculated. The bending lengths for the samples were between 1.3 and 1.7 cm (Table S2—Supporting Information). The bending lengths are comparable to previous reports using the heart loop method for fabrics. For example, a woven polyester fabric, Sukran reported a bending length of 1.36 cm, which validates our method. 

**Figure 3:**
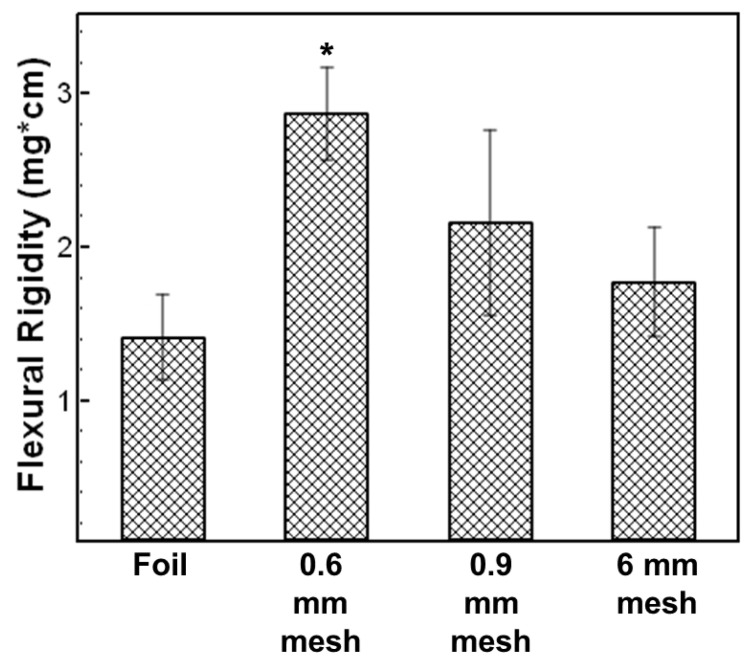
Flexural rigidity (a measure of stiffness) of the electrospun samples measured using the heart loop method. * indicates statistically different from sample spun on foil (no patterning) (*t* test, α = 0.05).

Interestingly, the highest bending length and resulting flexural rigidity was observed with the sample collected on the 0.6 mm mesh. There was a 2-fold increase in flexural rigidity using the 0.6 mm mesh compared to the sample collected on foil (Figure 3). The difference was statistically different as indicated by a student *t*-test (α = 0.05). We attribute this increase in rigidity to the density of the pattern. Notably, patterns from meshes with thicker wires that were less densely woven (e.g., 0.9 mm, 6 mm) were not as rigid as the 0.6 mm mesh with thinner wires that were more densely woven (i.e., 0.6 mm).

Overall, 0.9 mm and 6 mm mesh pattern collectors had enhanced rigidity and more uniform fibers compared to samples collected on foil. Further increasing the mesh size little advantage to using the mesh was observed. Thus, 0.6 mm mesh and 0.9 mm mesh were used for further experiments. 

Next, we investigated the effect of fiber patterning on the hydrophobicity of the samples. The contact angle was compared to specular, smooth polished nylon 6,6 with a reported water contact angle of 70° [36]. Upon electrospinning with patterning, the water contact angle was 133 ± 4° (using a 0.6 mm mesh) (Table 1), significantly higher than smooth nylon. Increasing the mesh size to 0.9 mm did not significantly affect the apparent water contact angle. Thus, the resulting patterned fibers were water repellent. The increase in contact angle has been attributed to the surface roughness of the fiber mat [37,38], specifically, hierarchical surface structure (micro- and nanoscale features) that mimics a lotus leaf with 3–10 µm protrusions and valleys decorated with 700–100 nm particles [39]. 

We note that the water contact angle of the patterned fibers was comparable to the electrospun fibers collected on foil, i.e., randomly deposited fibers with no pattern, with a water contact angle 137 ± 8° consistent with previous results for electrospun nylon [34,37,40]. This result suggests that the individual fiber-to-fiber spacing within the fiber mat is the most dominant spatial feature of the hierarchical surface roughness in the patterned samples compared to the millimeter features of the mesh. Overall, this result is promising for achieving water repellent fibers via electrospinning. 

**Figure 4:**
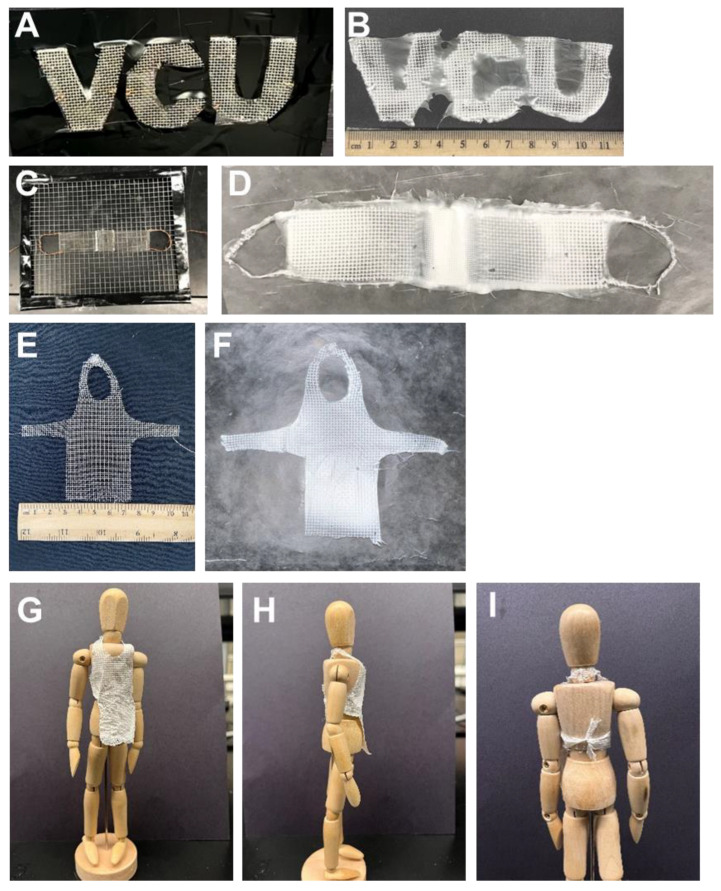
Representative functional patterns achieved by electrospinning onto 2D and 3D templates. Fiber patterning occurs during electrospinning. (**A**) Mesh collector formed into letters and (**B**) the resulting electrospun letters. (**C**) Mesh collector formed into a mask-like structure with ear loops and bridge for nose and (**D**) the resulting electrospun mask-like structure. (**E**) Apron mesh template, resulting electrospun apron (**F**), and electrospun apron on 8” mannequin: front view (**G**), side view (**H**), and rear view showing tie around the waist (**I**).

Building on our ability to electrospin patterned, water-repellent fibers, we next investigated direct fabrication of shapes of interest. The electrospun samples were observed to take the shape of the collector. By forming the metal mesh collectors into various 2D and 3D shapes (Figure 4A,C,E), functional patterns were produced. As a demonstration of the complexity of shapes that could be achieved, direct fabrication of letter-shaped mats (VCU) (Figure 4B) was done. The use of the letter-shaped collector facilitated preferential assembly of the patterned fibers over the letters due to electrostatic interactions [17]. Letter-shaped mats have been achieved previously using liquid collectors [18] or printed templates on an insulating substrate [17]. The letters using liquid collectors were approximately 5 mm × 5 mm; the letters using the printed templates were approximately 1 mm × 1 mm. The letters here are an order of magnitude larger and the fibers are simultaneously patterned during fiber processing. Additional functional shapes were produced including a mask-like structure with nose bridge and ear loops (Figure 4D) as well as an apron shape (Figure 4F). The resulting apron (Figure 4F) on an 8-inch mannequin is shown (Figure 4G–I). 

**Figure 5:**
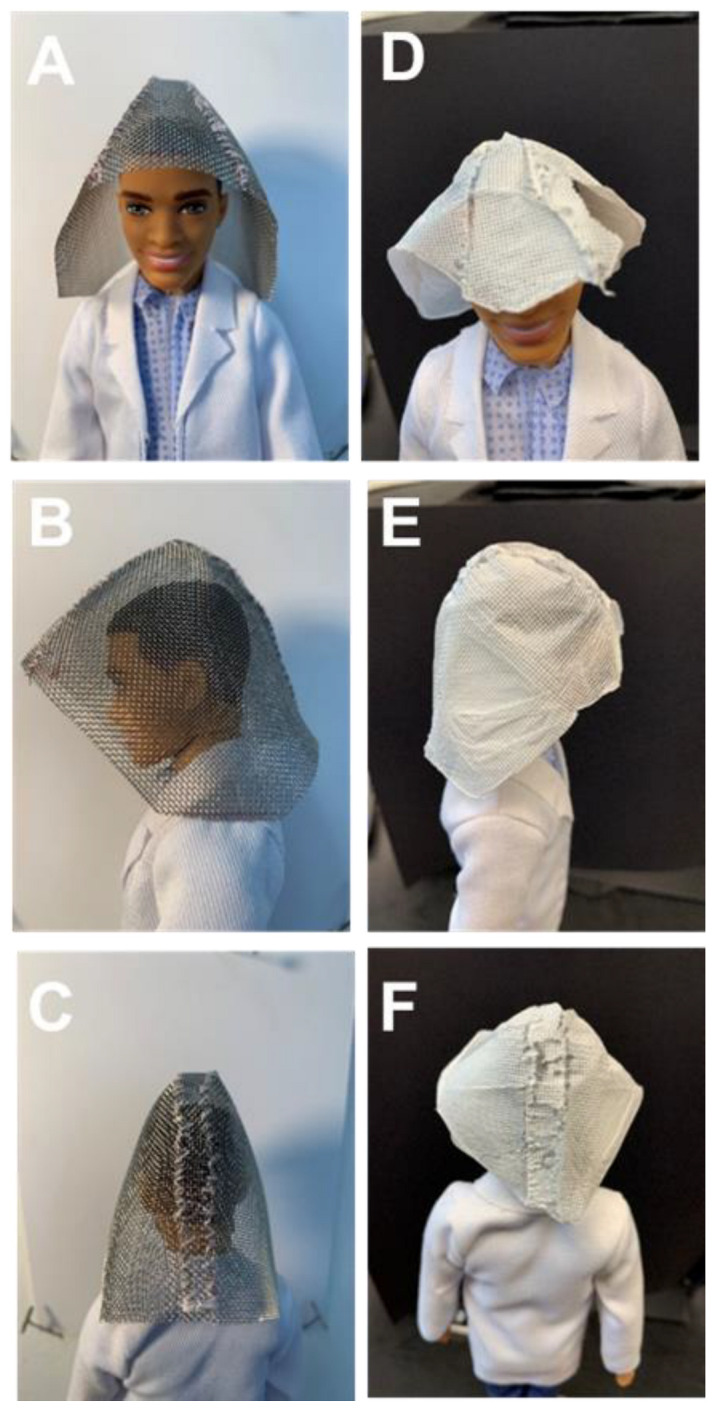
Representative functional patterns achieved by electrospinning onto 3D templates. Fiber patterning occurs during electrospinning. Mesh formed into a 3D hood template: front view (**A**), side view (**B**), rear view, and (**C**) resulting electrospun structure on a mannequin: front view (**D**), side view (**E**), and rear view (**F**).

The patterned fibers could also be formed into 3D templates. As proof-of-concept, mesh templates were sewn to form a hood (Figure 5A-C) and were directly covered by electrospinning. The resulting electrospun structure took the shape and structure (i.e., seams) of the template (Figure 5D–F). To cover the mesh hood, sections were covered, the mesh was rotated, and the subsequent section was covered. To cover the entire hood, it was oriented so that a side faced the spinneret and was well covered (electrospinning time 30 min). Next, the mesh was rotated so that the back of the hood faced the spinneret and was subsequently covered (electrospinning time 30 min). Finally, the hood was rotated so the remaining side of the hood faced the spinneret and was subsequently covered (electrospinning time 30 min). Visually, the mesh was uniformly covered and was removed from the template. The resulting electrospun structure took the shape and structure (i.e., seams) of the template (Figure 5D-F). Optical microscopy was performed on the different sections of the resulting hood (side, front, and back) (Supporting Information, Figure S3). No significant differences were observed in fiber patterning in the different sections of the hood; preferential assembly of the fibers along protrusions of the mesh relative to the voids was observed in all sections. Thus, this approach may be a useful tool for direct design and production of 3D items. 

Overall, we demonstrate one-step fabrication of water-repellent, 2D, and 3D functional patterned fibers by electrospinning onto patterned and shaped collector geometries. The fiber patterning increased the stiffness of the fiber mat compared to fibers collected on foil. Thus, we anticipate this as a simple, versatile tool for design and fabrication of 2D and 3D nanofiber-based items. In future work, it may be possible to scale up the size of the item or accelerate the production of custom item by combining the collectors used here with a high throughput electrospinning technique (e.g., multiple spinnerets or bubble electrospinning). Customizable, 3D electrospun products are being developed for clinical biomedical applications [41]. Potential applications may include personalized wound care and surgical meshes [28,29].

## 4. Conclusions

In this work, we demonstrate the ability to simultaneously pattern fibers and fabricate functional 2D and 3D shapes (e.g., mask like structures with nose bridges and ear loops, aprons, hoods) using a single-step electrospinning process. Using mesh templates, the electrospun fibers were preferentially attracted to the metal protrusions relative to the voids and the resulting macroscopic pattern and shape of the electrospun mat mimicked the template. On a microscopic scale, the electrostatic lensing effect of the collector decreased fiber diameter and narrowed the fiber size distribution. Notably, the patterning of the fibers also increased the rigidity of the fiber mat; there was a 2-fold increase in flexural rigidity using the 0.6 mm mesh compared to the sample collected on foil, improving the handle of electrospun materials. Overall, we anticipate that this approach will be a simple, versatile tool for design and fabrication of 2D and 3D nanofiber-based items with potential applications in personalized wound care and surgical meshes.

## Figures and Tables

**Table 1:** Water contact angle of selected patterned fiber samples compared to samples collected on foil as a measure of water repellent properties.

Sample	Contact Angle (°)	Reference
Smooth	70	[36]
Foil	137 ± 8	This work
0.6 mm mesh	133 ± 4	This work
0.9 mm mesh	134 ± 2	This work

## Data Availability

Not applicable.

## Supplementary Materials

The following supporting information can be downloaded at: https://www.mdpi.com/article/10.3390/polym15030533/s1, Figure S1: Overview of experimental portion of the heart loop method. Figure S2: Photographs of macroscopic appearance of fiber mats; Table S1: Effect of mesh size on fiber size and fiber size distribution; Table S2: Basis weight and bending length of electrospun samples. Figure S3: Representative optical microscopy images of various sections of the hood (front, side, back).
