# Supplementary material for: Direct Fabrication of Functional Shapes on 3D Surfaces Using Electrospinning
Source: Polymers (Basel). 2023 Jan 20;15(3):533. doi: 10.3390/polym15030533 (PMC9919392; doi:10.3390/polym15030533)
Supplement: Supplementary file 1 [file polymers-15-00533-s001.zip › polymers-2145063-supplementary.pdf]

Supporting Information

Direct Fabrication of Functional Shapes on 3D Surfaces Using Electrospinning

Ioana Caloian[1], Jocelyn Trapp[1], Melissa Williams[1], Ryan Kim[1], Mahmoud Moustafa[1] , E. Hawa Stwodah[2,], and Christina Tang[1,*]

1 Department of Chemical and Life Science Engineering, Virginia Commonwealth University, Richmond, VA

2 Department of Fashion Design and Merchandising, Virginia Commonwealth University, Richmond, VA

* Correspondence: ctang2@vcu.edu

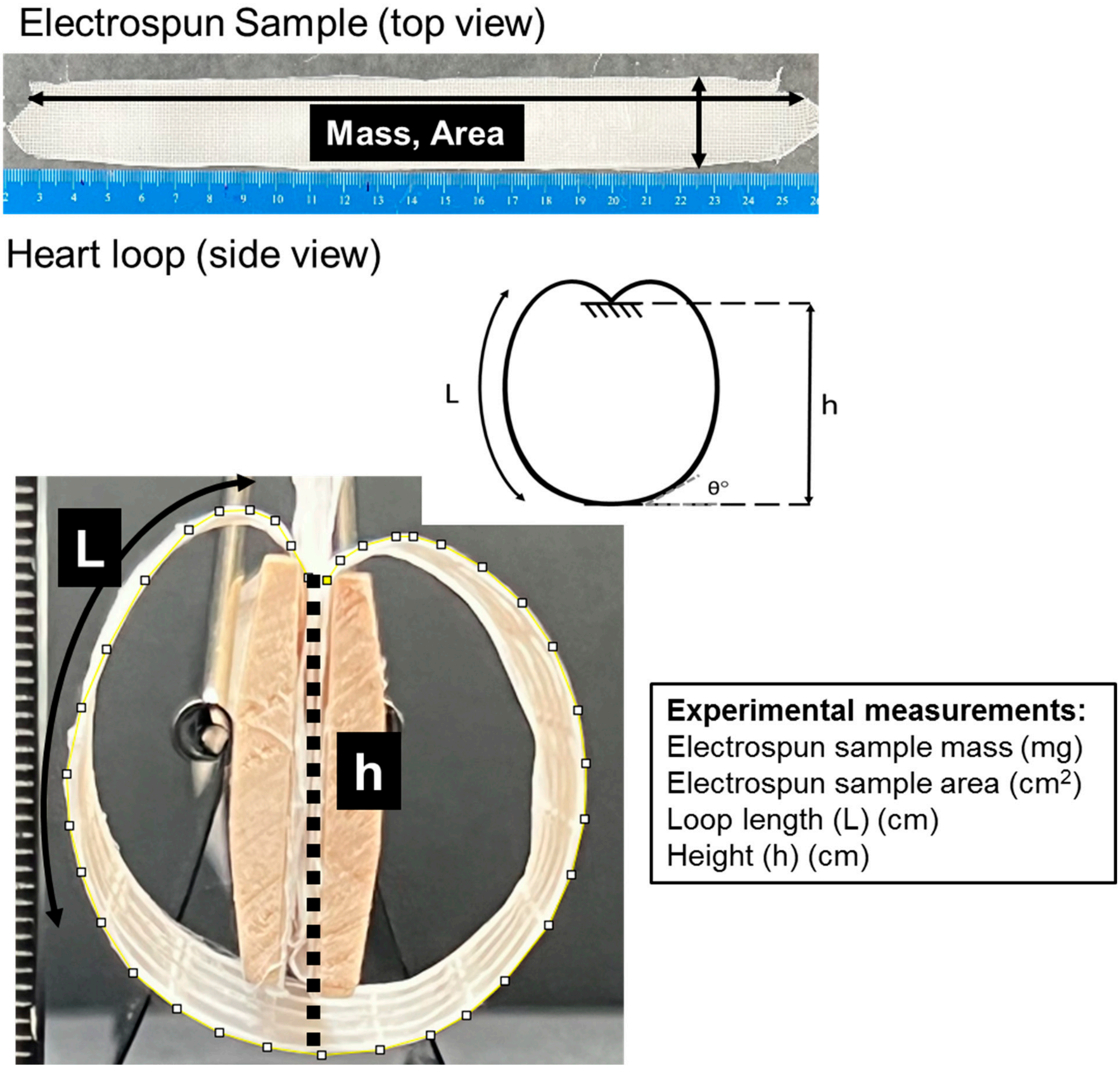

Figure S1. Overview of experimental portion of the heart loop method. Once the heart loop is made, the sample mass, area, loop length and loop height must be measured to calculated the bending length and flexural rigidity.

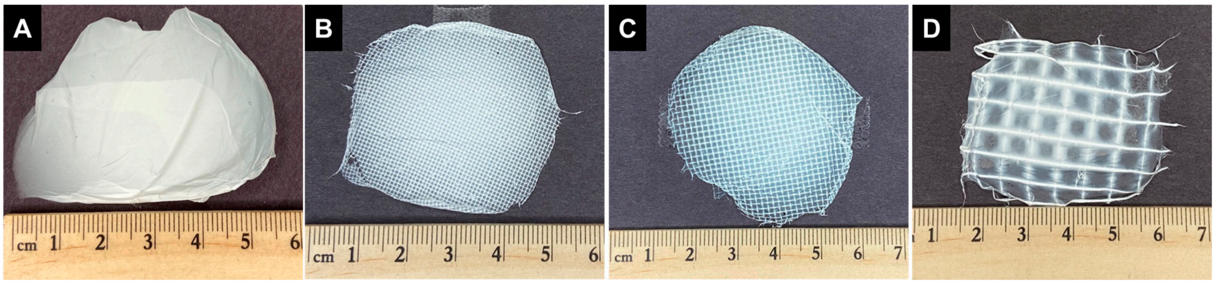

Figure S2. Photographs of macroscopic appearance of fiber mats spun on (A) foil, (B) 0.6 mm mesh, (C) 0.9 mm mesh and (D) 6 mm mesh. Since the electrospun fibers were preferentially attracted to the metal protrusions of the mesh relative to the voids, the macroscopic pattern of the electrospun mat mimics the woven mesh.

Table S1. Effect of mesh size on fiber size and fiber size distribution.

Sample	Fiber Diameter (nm)	Coefficient of Variation (%)
Foil	409±223	55
0.6 mm mesh	256±236	14
0.9 mm mesh	320±113	35
6 mm mesh	454±179	39

Table S2. Basis weight and bending length of electrospun samples

Sample	Basis Weight (g/m^2)	Bending Length (cm)	Flexural Rigidity (mg·cm)
Foil	4.8	1.4 ± 0.1	1.4±0.3
0.6 mm mesh	6.4	1.7± 0.1	2.9±0.3
0.9 mm mesh	6.9	1.5± 0.1	2.2±0.6
6 mm mesh	6.2	1.4 ± 0.1	1.8±0.4

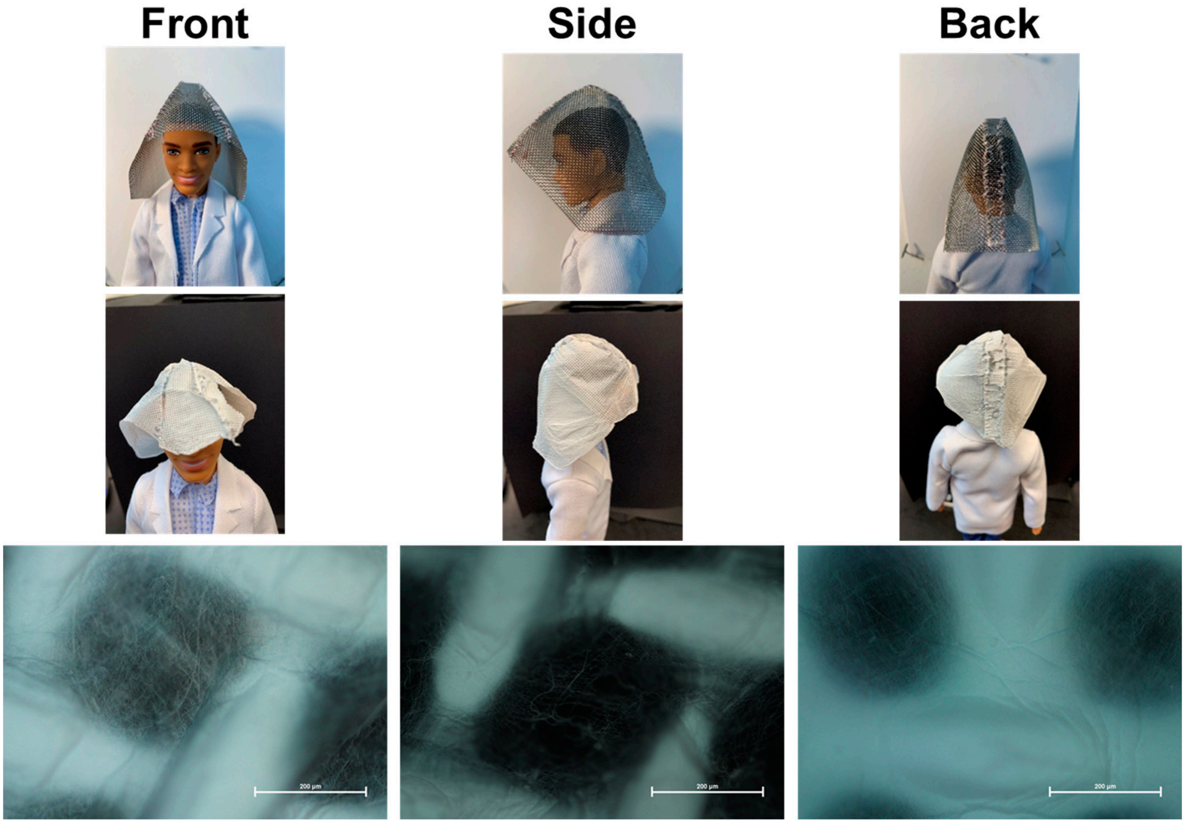

Figure S3. Representative optical microscopy images (polarized light, dark field), taken from various sections of the hood. Images from the front, side, back of the hood are shown. The scale bar is 200 microns.
